# *NR4A1* knockdown confers hepatoprotection against ischaemia‐reperfusion injury by suppressing TGFβ1 *via* inhibition of CYR61/NF‐κB in mouse hepatocytes

**DOI:** 10.1111/jcmm.16493

**Published:** 2021-05-03

**Authors:** Jun Cao, Ting Xu, Chengming Zhou, Shaochuang Wang, Baofei Jiang, Kun Wu, Long Ma

**Affiliations:** ^1^ Department of hepatic and Laparoscopic Surgery The First Affiliated Hospital of Xinjiang Medical University Urumqi China; ^2^ The Affiliated Huai’an No. 1 People’s Hospital of Nanjing Medical University Huai’an China; ^3^ The First Affiliated Hospital of Xinjiang Medical University Urumqi China; ^4^ Department of Hepatobiliary Surgery The Affiliated Huai’an No. 1 People’s Hospital of Nanjing Medical University Huai’an China; ^5^ Department of General surgery The Affiliated Huai’an No. 1 People’s Hospital of Nanjing Medical University Huai’an China; ^6^ Department of Intensive Care Unit The First Affiliated Hospital of Xinjiang Medical University Urumqi China

**Keywords:** cysteine‐rich angiogenic inducer 61, hepatic injury, ischaemia‐reperfusion, nuclear receptor subfamily 4, group A, member 1, nuclear‐factor kappa B, transforming growth factor β1

## Abstract

Nuclear receptor subfamily 4, group A, member 1 (NR4A1) can aggravate ischaemia‐reperfusion (I/R) injury in the heart, kidney and brain. Thus, the present study aimed to unravel the role of NR4A1 on hepatic I/R injury. For this purpose, the mouse hepatic I/R model and H/R‐exposed mouse hepatocytes model were established to stimulate the hepatic and hepatocellular damage. Then, the levels of ALT and AST as well as TNF‐α and IL‐1β expression were measured in the mouse serum and supernatant of hepatocyte s, respectively. Thereafter, we quantified the levels of NR4A1, CYR61, NF‐kB p65 and TGFβ1 under pathological conditions, and their interactions were analysed using ChIP and dual‐luciferase reporter gene assays. The in vivo and in vitro effects of NR4A1, CYR61, NF‐kB p65 and TGFβ1 on I/R‐induced hepatic and H/R‐induced hepatocellular damage were evaluated using gain‐ and loss‐of‐function approaches. NR4A1 was up‐regulated in the hepatic tissues of I/R‐operated mice and in H/R‐treated hepatocytes. Silencing NR4A1 relieved the I/R‐induced hepatic injury, as supported by suppression of ALT and AST as well as TNF‐α and IL‐1β. Meanwhile, NR4A1 knockdown attenuated the H/R‐induced hepatocellular damage by inhibiting the apoptosis of hepatocyte s. Moreover, we also found that NR4A1 up‐regulated the expression of CYR61 which resulted in the activation of the NF‐κB signalling pathway, thereby enhancing the transcription of TGFβ1, which was validated to be the mechanism underlying the contributory role of NR4A1 in hepatic I/R injury. Taken together, NR4A1 silencing reduced the expression of CYR61/NF‐κB/TGFβ1, thereby relieving the hepatic I/R injury.

## 1 INTRODUCTION

Ischaemia‐reperfusion (I/R) injury is a process that initiates blood supply limitation and subsequent blood flow reflux leading to the spread of innate immune responses and organ damage.^1^ I/R injury is considered as the major cause of hepatic injury during surgical operations such as hepatectomy and transplantation and remains the main cause of graft dysfunction after transplantation.^2^ Hepatic I/R injury is characterized by severe inflammation and extensive cell death.^3^, ^4^ Several groups of key molecules, such as microRNAs (miRNAs) and long non‐coding RNAs (lncRNAs), involved in hepatic pathways hold great potential for treating human liver I/R injury.^5^ However, the therapeutic roles of several pathways including NF‐κB, MAPK, JNK and Dusp14 have been investigated for the treatment of hepatic I/R.^6^ Characterization of the molecular mechanism associated with the pathophysiology of I/R could be beneficial to the development of novel therapeutic strategies against I/R‐induced tissue inflammation and organ dysfunction.^7^

Transcription factors are considered as crucial modulators of gene transcription with their implication in the progression of human diseases.^8^ Accordingly, a previous study about gene expression profiling by RNA sequencing in the mice model with hepatic I/R) has demonstrated a wide range of dysregulated molecules, including nuclear receptor subfamily 4, group A, member 1 (NR4A1), and cysteine‐rich angiogenic inducer 61 (CYR61).^9^ NR4A1, a transcription factor, belongs to the nerve growth factor‐induced gene B (NGFI‐B) family, which is induced in the context of hepatic I/R injury.^10^ More recently, NR4A1 has been reported to exert contributory effects on I/R‐induced injury in important organs such as heart and brain.^11^, ^12^ Genome‐wide expression analyses *via* Coexpedia and MEM database depicted that NR4A1was co‐expressed with CYR61 in the present study. CYR61 is a member of the CCN family of multifunctional proteins having pivotal roles in angiogenesis, inflammation and fibrous tissue repair.^13^ in the mice model with hepatic I/R) has demonstrated expression of CYR61 has been reported at inflammation and wound repair sites and proved to be also up‐regulated in pigs with intestinal I/R injury.^14^ Moreover, CYR61 regulates the cell viability in the pathogenesis of acute lymphoblastic leukaemia through the nuclear factor kappa B (NF‐κB) signalling pathway.^15^ Of note, NF‐κB is aberrantly expressed in I/R‐damaged liver tissues,^16^ and its activation is shown as a contributor of the aggravated hepatic I/R injury.^17^ Conversely, the inhibition of the NF‐κB pathway contributes to protection against hepatic I/R injury.^18^ Induction of TGFβ1 depends on the activation of NF‐κB and subsequent production of reactive oxygen species. Meanwhile, TGFβ1 contributes to the degradation of the NF‐κB inhibitor IκBα and promotes the nuclear translocation of the NF‐κB p65 subunit.^19^ TGFβ1 is considered as an effective inhibitor of cell growth targeting gene regulatory events ^20^ and has been proposed as a potential therapeutic target.^21^ Collectively, the above‐described findings indicated that the up‐regulation of TGFβ1 may potentially induce the reduction of apoptosis and oxidative damage in rats following I/R. Hence, the objective of the present study was to investigate the effects of NR4A1 on hepatocyte apoptosis and inflammation caused by hepatic I/R injury by regulating the CYR61/NF‐κB/TGFβ1 axis.

## 2 METHODS AND MATERIALS

### 2.1 Ethical statement

The animal experiments were approved by the Animal Ethics Committee of Xinjiang Medical University. The experiments involving animals were performed in line with the principles of Laboratory Animals of the National Institutes of Health.

### 2.2 Mouse I/R model

Twenty‐eight C57BL/6 male mice (aged 8‐10 weeks, weighting 21 ~ 28 g) were provided by the laboratory animal centre of Xinjiang Medical University. The mice model was constructed as previously described.^22^ Initially, 16 randomly selected mice were anaesthetized with 30 mg/kg tiletamine/zolazepam solution supplemented with 10 mg/kg xylazine. After midline laparotomy, the hepatic hilum was dissected and the first branch of the hepatic artery and portal vein was clamped by a microvascular forcep to supply the left and middle lobe of the liver. Meanwhile, the circulation in the cauda lobe was kept intact to prevent congestion in the intestinal vein. Afterwards, the peritoneum was closed with sterile saline gauze to prevent dehydration and the mice were placed on heating pads. After 90 minutes of hepatic ischaemia, the microvascular forcep was removed and the abdominal wall was closed with 6‐10 nylon sutures with the modelling success rate of 75% (12/16). Thereafter, the reperfusion was performed for about 6 hours, followed by anaesthesia and the whole blood was collected through the posterior orbital puncture, whereas liver tissue samples were collected for subsequent experimental analysis. The remaining 12 mice were sham‐operated and experienced the same procedures without vascular occlusion.

### 2.3 Construction of lentivirus particles

The sequence of NR4A1 was obtained from the NCBI database (https://www.ncbi.nlm.nih.gov/). The shRNA targeting NR4A1 (sh‐NR4A1) sequence was provided by Sigma Aldrich. (St. Louis, Missouri) and was ligated to the PLKO‐Puro vector (Sigma Aldrich). After sequencing, the target plasmids, that is NR4A1, psPAX2 and pMD2.G (Addgene, Cambridge, MA) were transduced into Human embryonic kidney 293 (HEK293T) cells. The cells were then seeded in a six‐well plate at a density of 3 × 10^5^ cells/well. After reaching 50%‐60% of cell confluence, the cells were infected with the supernatant of the culture medium containing lentiviral particles. Briefly, cells were infected two times with 1 μL lentivirus expressing negative control shRNA (sh‐NC) or shRNA against NR4A1 (sh‐NR4A1) at a titre of 5 × 10^8^ TU/ml.^23^ Thereafter, cells were cultured in a 5% CO_2_ cell incubator and harvested after 48 hours of transfection, followed by validation of transfection efficiency.

### 2.4 H/R modelling and cell transfection

Hepatocytes from mice were isolated using a modified two‐step collagenase perfusion of the mouse livers (H‐type; Roche Diagnostics, Mannheim, Germany).^24^ Besides, the plasmids expressing silenced a scramble siRNA (si)‐negative control (NC), si‐NR4A1 and si‐NF‐κB p65 were constructed, whereas overexpression plasmids such as overexpressed (oe)‐CYR61, oe‐TGFβ1 and oe‐NC were provided by GenePharma (Shanghai, China). Then, the isolated hepatocytes were cultured for 12 hours and the medium was renewed, followed by cell transfection with siRNAs or si‐NC using Lipofectamine 3000 (Invitrogen, Carlsbad, CA) for 48 hours. Afterwards, the hepatocytes were exposed to hypoxia (1% O_2_) for 12 hours, followed by reoxygenation (21% O_2_) for 2 hours. After transfection, the transfection efficiency was detected by the Reverse transcription‐quantitative polymerase chain reaction (RT‐qPCR).

48 h prior to modelling, the mice were infected two times with 1 μL lentivirus expressing negative control shRNA (sh‐NC) or shRNA against NR4A1 (sh‐NR4A1) at a titre of 5 × 10^8^ TU/ml.^23^

### 2.5 Computer‐based transcription factor‐mRNA analysis

Coexpedia (http://www.coexpedia.org/), an online co‐expression analysis website, was used to analyse gene co‐expression networks based on the microarray profiles of human and mouse samples from the GEO database. A web‐based multi‐experimental gene expression analysis was conducted and visualized using the MEM database (https://biit.cs.ut.ee/mem/index.cgi). Thereafter, the genes from 569 conditional human microarray sequence datasets regulated by transcription factors were analysed using the hTFtarget database (http://bioinfo.life.hust.edu.cn/hTFtarget). Moreover, JASPAR (http://jaspar.genereg.net/) database was utilized to obtained transcription factor binding profiles. ChIPBase database (http://rna.sysu.edu.cn/chipbase/) is an open database and used to study transcription factor binding sites and gene sequences.

### 2.6 RT‐qPCR

Total RNA was extracted as per the instructions of the TRIzol kit. The primers NR4A1, CYR61 and TGFβ1 were synthesized by Takara Bio, Inc (Otsu, Shiga, Japan) (Table 1). Then, the PrimeScript RT kit (RR036A, Takara) was applied to reversely transcribe RNA into cDNA. The real‐time PCR was conducted with the use of SYBR® Premix Ex TaqTM II Kit (RR820A, Takara) in an ABI 7500 quantitative PCR instrument (7500; Applied Biosystems, Foster City, CA). Afterwards, 2 μg of total RNA was taken as a template, whereas GAPDH was adopted as an internal reference. The relative expression level of NR4A1, CYR61 and TGFβ1 was calculated by the relative quantitative method (2^‐ △△ CT^ method). △△Ct = △Ct _model group_ ‐ △Ct _normal group_, △Ct = Ct _(target gene)_ ‐ Ct _(internal reference)_.

**TABLE 1:** RT‐qPCR primer sequences

Gene	Primer sequences
NR4A1	F: 5'‐TTGAGTTCGGCAAGCCTACC‐3'
	R: 5'‐GCACCACCACCCACGGAATCG‐3'
CYR61	F: 5'‐CGAGTTACCAATGACAACCCAG‐3'
	R: 5'‐TGCAGCACCGGCCATCTA‐3'
TGFβ1	F: 5'‐GCAAGTAAGCAGGAGCATTGCT‐3'
	R: 5'‐TGTCGGAAGTCGATAAGCG‐3'
GAPDH	F: 5'‐CACTGAGCAAGAGAGGCCCTAT‐3'
	R: 5'‐GCAGCGAACTTTATTGATGGTATT‐3'

Abbreviations: NR4A1, nuclear receptor subfamily 4, group A, member 1; CYR61, cysteine‐rich angiogenic inducer 61; TGFβ1, transforming growth factor β1; GAPDH, glyceraldehyde‐3‐phosphate dehydrogenase; F, forward; R, reverse.

### 2.7 Western blot analysis

Phenylmethylsulfonyl fluoride (PMSF)‐containing radioimmunoprecipitation assay (RIPA) lysis buffer (R0010; Solarbio Science and Technology Ltd., Beijing, China) was added to lyse the collected cell pellet. Cells were incubated on ice for 30 minutes, centrifuged at 12,000 g and 4°C for 10 minutes. Protein (50 μg) was separated by 10% sodium dodecyl sulphate‐polyacrylamide gel electrophoresis and then transferred to a polyvinylidene fluoride (PVDF) membrane. After blocking with 5% skimmed milk powder for 1 hours, the membrane was washed with TBST 3 times for 5 minutes each time. Subsequently, PVDF membrane were co‐cultured with diluted antibodies to NR4A1 (ab232375; 1:1000, Abcam, Cambridge, UK), CYR61 (1 µg/mL, ab24448; Abcam), NFκB p65 (ab19870; 1 µg/mL, Abcam), NF‐kB p65 (phospho S536) (sc‐52893; 1 µg/mL; Abcam), TGFβ1 (sc‐52893; 1:1000, Santa Cruz Biotechnology, Inc, Santa Cruz, CA) and glyceraldehyde‐3‐phosphate dehydrogenase (GAPDH; ab8245; 1:1000, Abcam) at 4°C overnight. The membrane was incubated with 1:100 diluted horseradish peroxidase (HRP)‐labelled secondary goat antirabbit immunoglobulin G (IgG) antibody (HA1003; Yanhui Biotechnology Co., Ltd. Shanghai, China) for 1 hours. Later, the membrane was reacted with electrochemiluminescence (ECL) solution (ECL808‐25; Biomiga. Inc San Diego) for 1 minutes at room temperature. The liquid was aspirated and ECL luminescent solution was used for development in the gel imager. The relative expression of the protein was expressed by the ratio of the grey value of the target band to the internal reference band GAPDH.

### 2.8 Dual‐luciferase reporter gene assay

Wild‐type and mutant CYR61 and TGFβ1 reporter plasmids (wt‐CYR61, mut‐CYR61, wt‐TGFβ1, mut‐TGFβ1) were designed and provided by Shanghai GenePharma Co., Ltd (Shanghai, China). The cells were co‐transfected with oe‐NC/oe‐NR4A1 and wt‐CYR61/mut‐CYR61, or co‐transfected with oe‐NC/oe‐p65 and wt‐TGFβ1/mut‐TGFβ1 into HEK‐293T cells for culture for 48 hours. Changes in luciferase activity were detected using Genecopoeia dual‐luciferase assay kit (D0010; Beijing Solarbio science & technology co., Ltd.). The luminance was detected on GLomax20/20 Luminometer (E5311; Shaanxi Zhongmei Biotechnology Co., Ltd., Shaanxi, China).

### 2.9 Chromatin immunoprecipitation (ChIP) assay

The cells were cross‐linked with 16% formaldehyde and lysed with cell lysis buffer followed by sonication. Thereafter, antibodies NR4A1 (ab232375; 1:1000, Abcam) and p65 (ab19870; 1:1000, Abcam) were added into the cell lysate for overnight incubation. After overnight incubation, cells were incubated with magnetic beads to capture protein‐DNA complex, which was eluted. Then, 5 mmol/L NaCl was added to reverse the crosslinking. After crosslinking, the protein‐DNA complex was harvested, and the relative expression of CYR61 and TGFβ1 in the complex was determined by RT‐qPCR.

### 2.10 Biochemical analysis and Enzyme‐linked immunosorbent assay (ELISA)

The levels of alanine aminotransferase (ALT) and aspartate aminotransferase (AST) in mouse serum and cell supernatant were evaluated using the ALT (c009‐2‐1) and AST (c010‐2‐1) detection kits from NanJing JianCheng Bioengineering Institute (Nanjing, China) strictly in accordance with the protocol. The contents of TNF‐α and IL‐1β levels in serum and cell supernatant were examined using ELISA kits from Mskbio (Wuhan, China) (TNF‐α: 69‐99985; IL‐1β: 69‐59812). Briefly, the samples were centrifuged at 4000 rpm/min for 10 minutes at 4°C, followed by the collection of the supernatant. Then, the standard samples were added with 2 mL of distilled water to prepare a 20 ng/mL standard sample solution. For this purpose, 8 standard tubes were set, in which the first tube was added with a 900 μL diluted sample solution, whereas the rest of the tubes were added with a 500 μL sample solution. The content in each tube was repeatedly diluted with the eighth tube set as a blank control. Then, each well was added with 100 μL standard or test samples and placed on the reaction place at 37℃ for 120 minutes. Each sample was detected following the instructions of the ELISA kit. The corresponding IL‐9 content was determined on the curve based on the sample optical density (OD) value.

### 2.11 Haematoxylin and eosin (HE) staining

Liver tissues were fixed in 10% neutral formalin for 24 hours, dehydrated with gradient alcohol and cleared in xylene. The tissues were then embedded with paraffin and sliced into sections. The sections were hydrated with alcohol in a gradient manner followed by washing with distilled water for 1 minutes. Afterwards, the sections were stained with haematoxylin for 3 minutes and washed by tap water. Then, the sections were differentiated in 0.5% hydrochloric acid alcohol for 10 seconds and blued for 10 minutes followed by eosin staining for 5 minutes. Finally, the tissues were conventionally dehydrated and sealed with neutral gum. Each section was observed under an optical microscope (XP‐330, Shanghai Bingyu Optical Instrument Co., Ltd., Shanghai, China). A pathologist who was blinded to group identity and rated the extent of sinusoidal congestion, vacuolization/ballooning and necrosis on a scale from 0 to 4 according to the Suzuki classification.^25^

### 2.12 Terminal deoxynucleotidyl transferase‐mediated dUTP nick end‐labelling (TUNEL) staining

Paraffin‐embedded sections were dewaxed, hydrated and then immersed in 3% H_2_O_2_ for 12 minutes. The sections were then incubated with proteinase‐K (20 μg/ml in Tris/HCl) at room temperature for 30 minutes. After 4',6‐diamidine‐2‐phenylindole (DAPI) staining, the cell apoptosis was observed and images were captured under a fluorescence microscope (Eclipse Ti, Nikon, Tokyo, Japan).

### 2.13 Immunohistochemical staining (IHC)

Paraffin‐embedded sections were routinely dewaxed with xylene and hydrated in gradient alcohol, followed by phosphate‐buffered saline (PBS) washing. Next, the sections were soaked in 3% H_2_O_2_ for 10 minutes, followed by PBS washing and antigen retrieval. Afterwards, the sections were blocked with 5% bovine serum albumin (BSA) at 37°C for 30 minutes. The sections were then incubated in 50 μL of rabbit antimouse NR4A1 (ab232375; 1:100, Abcam) overnight at 4°C, and rinsed with PBS for 2 minutes. Thereafter, the sections were incubated with 50 μL biotinylated goat antimouse IgG (RXE0155, Shanghai Rongchuang Biotechnology Co., Ltd., Beijing, China) (1:100) at 37°C for 30 minutes. After strept‐avidin‐biotin complex (SAB) staining, the sections were developed with diaminobenzidine (DAB) and counterstained with haematoxylin for 5 minutes. PBS buffer served as negative control instead of the primary antibody. The rate of positive cells (brown‐yellow) more than 10% was considered as positive staining, and the staining was mainly located in the cytoplasm or cell membrane. Five high‐power fields were randomly selected. The positive rate was scored proportionally with the area of immunopositive staining (0%, 0 point; 1%‐25%, 1 point; 26%‐50%, 2 points; 51%‐75%, 3 points; 76%‐100%, 4 points) multiplied by the intensity staining (negative, 0 point; weak positive, 1 point; moderate positive, 3 points; 3, strongly positive, 4 points). The scores were independently given by two pathologists.

### 2.14 Cell Counting kit‐8 (CCK‐8) assay

After 48 hours of infection, cells in the logarithmic growth phase were dispersed into cell suspension using Dulbecco's Modified Eagle's Medium (DMEM) containing 10% foetal bovine serum (FBS). Then, 1 × 10^4^ /mL cell suspension was seeded into 96‐well plate, with 8 parallel wells, 100 μL per well, and cultured in a 37°C, 5% CO_2_ cell incubator. The cells were culture at and collected at different time points 24, 48 and 72 hours, respectively, followed by incubation with 10 µL CCK8 (Sigma) for 1 hours, whereas absorbance (OD) of each well was measured at 450 nm on a microplate reader (NYW‐96 M, Beijing Noahway Instruments Co., Ltd.).

### 2.15 Flow cytometry

Cells were seeded in 96‐well plates, 2.0 × 10^3^ cells per well and washed twice in PBS solution. After centrifugation, the cells were resuspended in 200 μL of binding buffer. Then, the cells were reacted with 10 μL of Annexin V‐fluorescein isothiocyanate (FITC) (ab14085; Abcam) and 5 μL of propidium iodide (PI) for 15 minutes at room temperature in the dark. Additionally, the cells were rinsed in 300 μL of the binding buffer after which the apoptosis was detected on a Guava Easycyte flow cytometer (Millipore, Billerica, MA, USA) at an excitation wavelength of 488 nm.

### 2.16 Statistical analysis

All data were expressed as mean ± standard deviation and analysed by SPSS 22.0 statistical software (IBM Corp., Armonk, New York). The unpaired data between two groups were compared using unpaired t test. One‐way analysis of variance (ANOVA) was conducted for comparison among multiple groups, followed by Tukey's post hoc test. Data of different groups at different time points were compared by repeated measures ANOVA, followed by Bonferroni's post hoc test. A *P* <.05 represented statistical significance.

## 3 RESULTS

### 3.1 NR4A1 was highly expressed in liver tissues of mice with hepatic I/R injury

**FIGURE 1:**
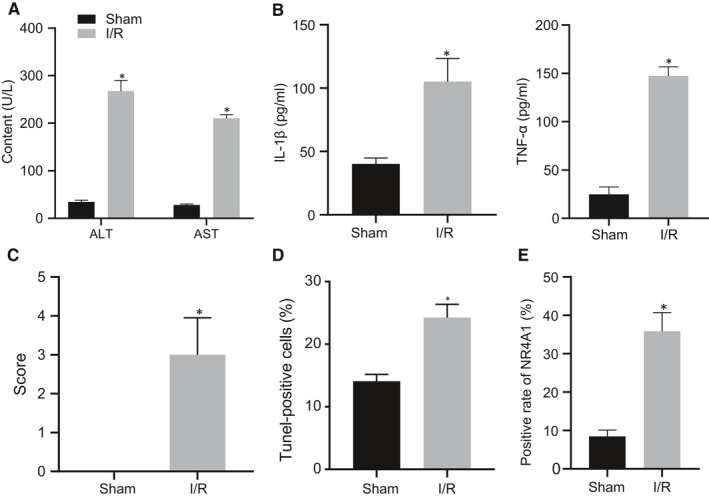
The expression of NR4A1 is up‐regulated in hepatic tissues of mice with hepatic I/R injury. A, ALT and AST levels in serum of mice were tested by biochemical analysis. ^*^ *P* < 0.05 *vs*. sham‐operated mice. B, Serum levels of TNF‐α and IL‐1β were detected by ELISA. ^*^ *P* < 0.05 *vs*. sham‐operated mice. C, Hepatic injury was observed after HE staining. D, Cell apoptosis in liver tissues was detected using TUNEL. E, NR4A1 positive rate was evaluated by IHC staining. ^*^ *P* < 0.05 *vs*. sham‐operated mice. The values were the measurement data, expressed as mean ± standard deviation, and the unpaired t test was used between the two groups, n = 12

Initially, a mouse model of hepatic I/R injury was established and the serum levels of ALT and AST in mice were measured to identify the liver injury after modelling. We found that the levels of ALT and AST in I/R mice were significantly higher than those in sham‐operated mice (Figure 1A) (*P* < 0.05). Besides, the serum expression of TNF‐α and IL‐1β was examined using ELISA, which showed that TNF‐α and IL‐1β contents in I/R mice serum were notably increased than that in sham‐operated mice (Figure 1B) (*P* < 0.05). HE staining exhibited hepatocyte oedema in some areas, flaky necrosis, massive infiltration of neutrophils and disappearance of hepatic sinus structure in liver tissues of mice experienced I/R treatment (Figure 1C). Moreover, TUNEL staining was employed to detect the apoptosis of liver cells and the results of TUNEL staining and DAPI fluorescence staining exhibited significantly increased apoptosis in I/R mice than that in untreated mice (Figure 1D). Thereafter, the positive expression of NR4A1 in liver tissues was evaluated using IHC staining which displayed that NR4A1 positive expression in I/R mice was markedly up‐regulated than that in untreated mice (Figure 1E) (*P* <.05). Taken together, NR4A1 was highly expressed in liver tissues of mice with hepatic I/R injury.

### 3.2 Hypoxia/reoxygenation‐exposed hepatocytes exhibit high expression of NR4A1

**FIGURE 2:**
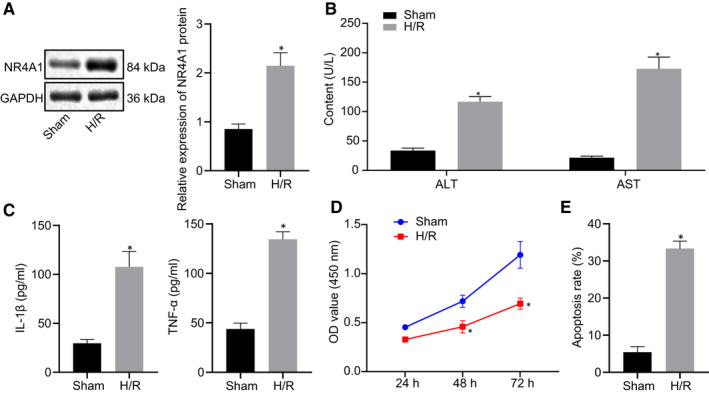
NR4A1 expression is increased in H/R‐treated hepatocytes. A, The protein expression of NR4A1 in H/R‐treated hepatocytes and control hepatocytes measured using Western blot analysis. B, ALT and AST levels in hepatocyte supernatant measured by biochemical analysis. C, contents of TNF‐α and IL‐1β in H/R‐treated hepatocytes and control hepatocytes detected by ELISA. D, The viability of H/R‐treated hepatocytes and control hepatocytes in mice tested by CCK‐8. E, The apoptosis of H/R‐treated hepatocytes and control hepatocytes detected by flow cytometry. ^*^ *P* < 0.05 *vs*. the control hepatocytes. The values were the measurement data, expressed as mean ± standard deviation, and the unpaired t test was used between the two groups, and data at different time points were analysed using repeated measures ANOVA, followed by Bonferroni post hoc test

Hepatocytes were isolated from sham‐operated mice and cultured under H/R conditions. The protein expression of NR4A1 in hepatocytes was examined using Western blot analysis, which revealed that the protein expression of NR4A1 in H/R‐treated hepatocytes was higher than that in the control hepatocytes (*P* < 0.05) (Figure 2A). Next, ALT and AST levels in the supernatant of mouse hepatocytes were determined using biochemical analysis, which showed that ALT and AST levels in H/R‐treated hepatocytes were remarkably increased as compared to the control hepatocytes (*P* < 0.05) (Figure 2B). As reflected by ELISA, the contents of TNF‐α and IL‐1β in H/R‐treated hepatocytes were higher than that in the control hepatocytes (*P* < 0.05) (Figure 2C). Besides, our data from cellular function assessment by CCK‐8 and flow cytometry exhibited a striking reduction in cell viability of H/R‐treated hepatocytes, whereas apoptosis was significantly increased in comparison with the control hepatocytes (*P* < 0.05) (Figure 2D, 2E). Collectively, hypoxia/reoxygenation enhanced the expression of NR4A1 in hepatocytes.

### 3.3 Knockdown of NR4A1 attenuates H/R‐induced injury in hepatocytes

**FIGURE 3:**
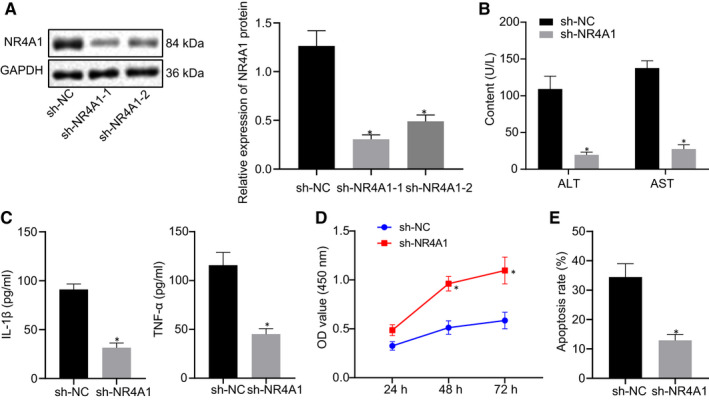
Silencing of NR4A1 relieves H/R‐induced hepatic injury. A, The protein expression of NR4A1 in the H/R‐treated hepatocytes transfected with si‐NR4A1 measured using Western blot analysis. B, ALT and AST levels in the supernatant of H/R‐treated hepatocytes upon NR4A1 silencing. C, The contents of TNF‐α and IL‐1β in H/R‐treated hepatocytes detected by ELISA upon NR4A1 silencing. D, Viability of H/R‐treated hepatocytes monitored by CCK‐8 upon NR4A1 silencing. E, Apoptosis of H/R‐treated hepatocytes evaluated by flow cytometry upon NR4A1 silencing. ^*^ *P* < 0.05 *vs*. si‐NC‐transfected cells. All data were expressed as mean ± standard deviation. An unpaired t test was performed for comparisons of unpaired data between the two groups. One‐way ANOVA was conducted for comparison among multiple groups, followed by Tukey's post hoc test. Data of different groups at different time points were compared by repeated measures ANOVA, followed by Bonferroni's post hoc test. The cell experiment was repeated three times

H/R‐treated hepatocytes were then transfected with siRNA against NR4A1 (si‐NR4A1), and the transfection efficiency in hepatocytes was validated by Western blot analysis. Notably, our data exhibited that the protein expression of NR4A1 in cells transfected si‐NR4A1‐1 and si‐NR4A1‐2 was markedly declined in comparison with the cells transfected with si‐NC, which verified the silencing efficiency (Figure 3A). Moreover, si‐NR4A1‐1 with better silencing efficacy was selected for subsequent experiments (*P* < 0.05). As expected, ALT and AST levels (Figure 3B), as well as contents of TNF‐α and IL‐1β (Figure 3C), were remarkably reduced upon NR4A1 silencing (*P* < 0.05). Meanwhile, the viability of hepatocytes (Figure 3D) was potentiated but the apoptosis (Figure 3E) was inhibited by NR4A1 silencing (*P* < 0.05). Coherently, knockdown of NR4A1 could potentially reduce the H/R‐induced injury in hepatocytes.

### 3.4 Inhibition of NR4A1 alleviates H/R‐induced hepatocyte injury through suppressing CYR61

**FIGURE 4:**
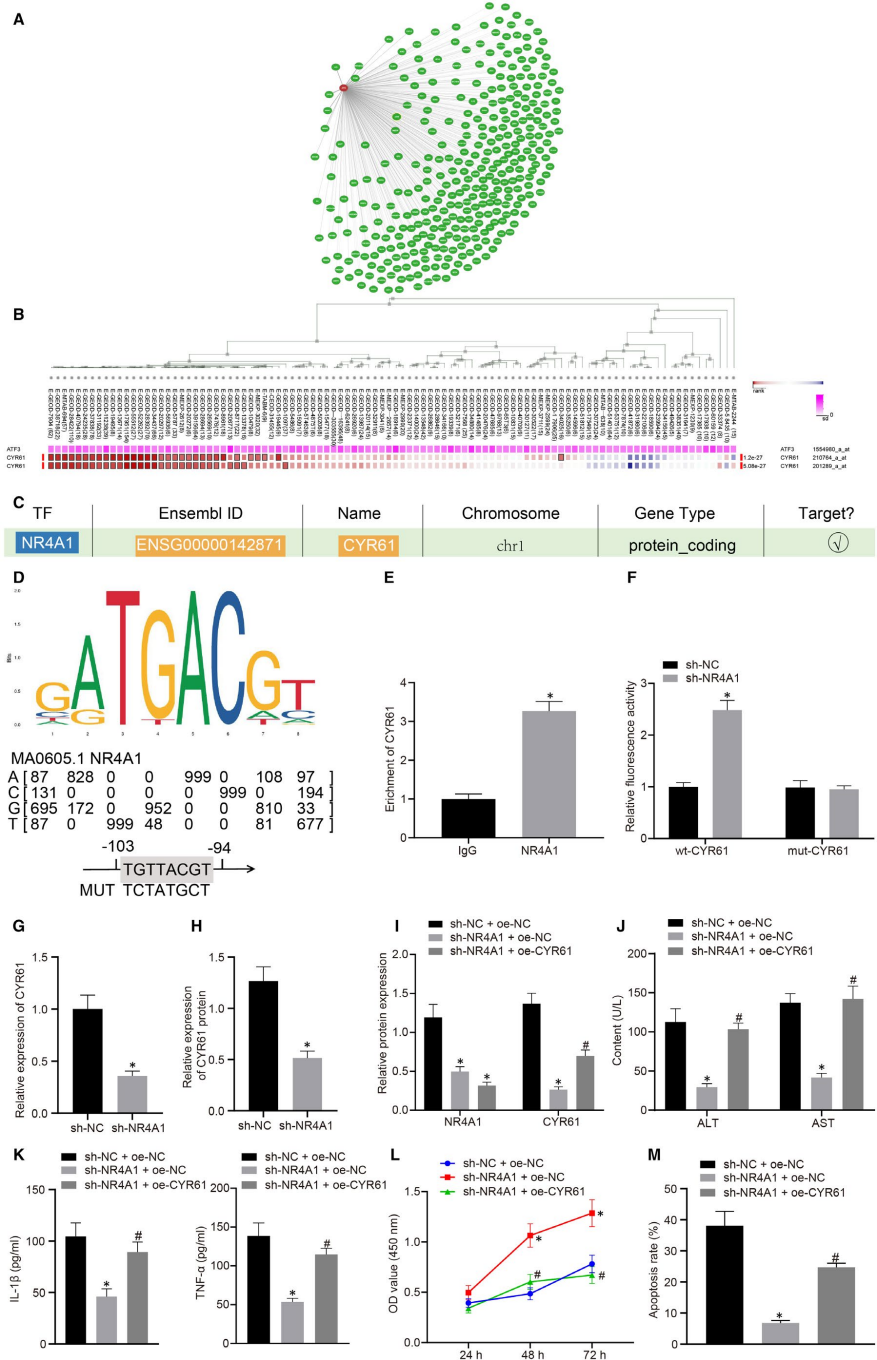
NR4A1 loss‐of‐function relieves H/R‐induced hepatocyte injury *via* suppression of CYR61 expression. A, NR4A1 co‐expressed genes predicted by Coexpedia. The closer location of the gene in the cycle to NR4A1 indicates a closer relationship. B, The co‐expression relationship between NR4A1 and CYR61 analysed by the MEM database, *P* = 1.17e‐15. C, The binding relationship between NR4A1 and CYR61 verified using the hTFtarget website. D, The binding region of NR4A1 in CYR61 promoter in mice predicted by the JASPAR website. E, The binding of NR4A1 to CYR61 promoter validated using ChIP assay. ^*^ *P* < 0.05 *vs*. IgG. F, The relationship between NR4A1 and CYR61 identified by dual‐luciferase reporter assay. ^*^ *P* < 0.05 *vs*. oe‐NC‐transfected cells. G, The mRNA expression of CYR61 determined using RT‐qPCR. ^*^ *P* < 0.05 *vs*. si‐NC‐transfected cells. H, The protein expression of NR4A1 determined by Western blot analysis. ^*^ *P* < 0.05 *vs*. si‐NC‐transfected cells. I, The protein expression of NR4A1 and CYR61 was measured by the means of Western blot analysis. J, ALT and AST levels in the supernatant of H/R‐treated hepatocytes detected by biochemical analysis. K, The levels of TNF‐α and IL‐1β in the H/R‐treated hepatocytes detected by ELISA. L, The viability of H/R‐treated hepatocytes assessed using CCK‐8. M, The apoptosis of H/R‐treated hepatocytes tested by flow cytometry. In panels I‐M, ^*^ *P* < 0.05 *vs*. cells co‐transfected with si‐NC and oe‐NC. ^#^ *P* < 0.05 *vs. c*ells co‐transfected with si‐NR4A1 and oe‐NC. All data were expressed as mean ± standard deviation. An unpaired t test was performed for comparisons of unpaired data between the two groups. One‐way ANOVA was conducted for comparison among multiple groups, followed by Tukey's post hoc test. Data of different groups at different time points were compared by repeated measures ANOVA, followed by Bonferroni's post hoc test. The cell experiment was repeated three times

Our further findings from the Coexpedia database analysis revealed that CYR61 ranked 12th among its co‐expressed genes (Figure 4A, Table 2). Furthermore, the co‐expression of NR4A1 and CYR61 was confirmed by the MEM database analysis (Figure 4B). Besides, previously reported experimental data has indicated the up‐regulation of both CYR61 and NR4A1 in the hepatic I/R model, and CYR61 has been suggested as a hub gene in hepatic I/R injury.^9^, ^10^ Accordingly, our results through the hTFtarget database reported a binding relationship between NR4A1 and CYR61 (Figure 4C). The binding region of NR4A1 in mouse CYR61 promoter was predicted by the JASPAR database (Figure 4D). Moreover, the ChIP assay further validated the enrichment of CYR61 by demonstrating that CYR61 was coprecipitated with NR4A1 as compared to IgG (*P* < 0.05) (Figure 4E). The subsequent dual‐luciferase reporter assay in HEK‐293T cells also suggested that co‐transfection with oe‐NR4A significantly enhanced the luciferase activity of wt‐CYR61 (*P* < 0.05). However, no significant change was observed in luciferase activity of mut‐CYR61 (*P* > 0.05) (Figure 4F). Thereafter, the expression of CYR61 in H/R‐treated hepatocytes transfected with si‐NR4A1 was measured using RT‐qPCR and Western blot analysis. The results displayed that CYR61 at mRNA and protein levels in H/R‐treated hepatocytes were notably reduced by NR4A1 silencing (*P* < 0.05) (Figure 4G, 4H). However, the reduced expression of CYR61 by si‐NR4A1 in hepatocytes was restored by the co‐transfection with oe‐CYR61 (*P* < 0.05) (Figure 4I). Thus, we further examined the role of H/R‐treated hepatocytes after co‐transfection with oe‐CYR61 and si‐NR4A1. The obtained data suggested that the reduction of ALT and AST levels (Figure 4J), as well as TNF‐α and IL‐1β levels (Figure 4K) in H/R‐treated hepatocytes caused by NR4A1 silencing, was reversed by restoration of CYR61 (*P* < 0.05). Moreover, our results from CCK‐8 and flow cytometry exhibited that the contributory effect of NR4A1 silencing on the viability of H/R‐treated hepatocytes (Figure 4L) and its suppressive effects on apoptosis (Figure 4M) was diminished by CYR61 overexpression (*P* < 0.05). The above‐mentioned findings revealed that NR4A1 knockdown relieved the H/R‐induced hepatocyte injury *via* suppression of CYR61.

**TABLE 2:** The top 24 co‐expressed genes with the higher score obtained from Coexpedia

Rank	Gene	Score	Rank	Gene	Score
1	NR4A2	85.312	13	NDRG1	20.562
2	NR4A3	63.466	14	SLC2A3	18.864
3	FOSB	60.663	15	USP36	18.087
4	ZFP36	56.701	16	GADD45B	15.607
5	NR4A1	47.924	17	CDKN1A	15.459
6	FOSL2	29.901	18	IER2	14.262
7	EGR1	29.85	19	CSRNP1	13.833
8	JUNB	28.852	20	SGK1	13.056
9	FOS	26.632	21	CYR61	12.843
10	EGR3	26.358	22	BTG2	12.777
11	HBEGF	23.482	23	BHLHE40	12.263
12	DUSP1	20.701	24	PPP1R15A	11.402

### 3.5 CYR61 exaggerates H/R‐induced hepatocyte injury *via* NF‐κB signalling pathway

**FIGURE 5:**
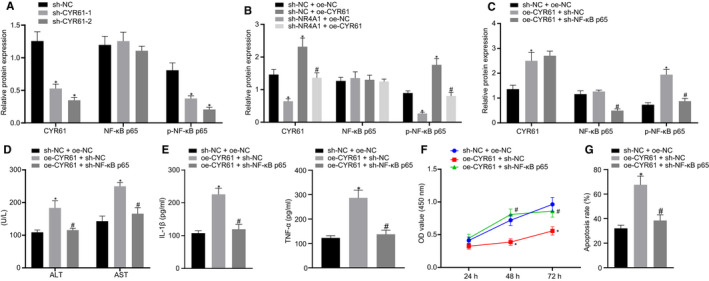
Overexpression of CYR61 aggravates the H/R‐induced hepatocyte injury through activating the NF‐κB signalling pathway. A, The protein expression of CYR61, NF‐κB p65 and p‐NF‐κB p65 in H/R‐exposed hepatocytes transfected with si‐CYR61 measured by Western blot analysis. ^*^ *P* < 0.05 *vs*. si‐NC‐transfected cells. B, The protein expression of CYR61, NF‐κB p65 and p‐NF‐κB p65 in H/R‐exposed hepatocytes co‐transfected with si‐NR4A1 and oe‐CYR61 measured by Western blot analysis. ^*^ *P* < 0.05 *vs*. cells co‐transfected with si‐NC and oe‐NC. ^#^ *P* < 0.05 *vs*. cells co‐transfected with si‐NR4A1 and oe‐NC. C, The protein expression of CYR61, NF‐κB p65 and p‐NF‐κB p65 in H/R‐exposed hepatocytes co‐transfected with oe‐CYR61 and si‐NF‐κB p65 measured by Western blot analysis. D, The AST and ALT levels in H/R‐exposed hepatocytes co‐transfected with oe‐CYR61 and si‐NF‐κB p65 examined using biochemical analysis. E, The levels of TNF‐α and IL‐1β in H/R‐exposed hepatocytes co‐transfected with oe‐CYR61 and si‐NF‐κB p65 detected by ELISA. F, Viability of H/R‐exposed hepatocytes co‐transfected with oe‐CYR61 and si‐NF‐κB p65 evaluated by CCK‐8. G, Apoptosis of H/R‐exposed hepatocytes co‐transfected with oe‐CYR61 and si‐NF‐κB p65 assayed by flow cytometry. In panels C‐F, ^*^ *P* < 0.05 *vs*. cell co‐transfected with si‐NC and oe‐NC. ^#^ *P* < 0.05 *vs*. cell co‐transfected with oe‐CYR61 and si‐NC. All data were expressed as mean ± standard deviation. An unpaired t test was performed for comparisons of unpaired data between two groups. One‐way ANOVA was conducted for comparison among multiple groups, followed by Tukey's post hoc test. Data of different groups at different time points were compared by repeated measures ANOVA, followed by Bonferroni's post hoc test. The cell experiment was repeated three times

Previously reported experimental results have shown the potential of CYR61 to promote the activation of the NF‐κB signalling pathway,^15^, ^26^, ^27^ whereas NF‐κB activation has been indicated to aggravate the hepatic I/R injury.^17^ Therefore, we hypothesized that CYR61 might regulate the I/R‐induced hepatocyte injury *via* the NF‐κB signalling pathway. For this purpose, the H/R‐exposed hepatocytes were transfected with si‐CYR61. Notably, our data exhibited higher silencing efficiency of si‐CYR61‐2 (*P* < 0.05); therefore, si‐CYR61‐2 was selected for subsequent experiments. Furthermore, Western blot analysis was performed to investigate the expression of NF‐κB p65 and phosphorylation of NF‐κB p65 proteins in H/R‐exposed hepatocytes. We found that inhibition of CYR61 triggered the significant reduction in protein expression of CYR61 and phosphorylation of NF‐κB p65 (*P* < 0.05) (Figure 5A). Furthermore, the H/R‐exposed hepatocytes were co‐transfected with si‐NR4A1 and oe‐CYR61. The protein expression of CYR61 and NF‐κB p65 phosphorylation level in the H/R‐exposed hepatocytes were notably reduced by the silencing of NR4A1, but elevated by the overexpression of CYR61. However, the reduction in NF‐κB p65 phosphorylation by si‐NR4A1 was counteracted by the restoration of CYR61 (Figure 5B). Additionally, hepatocytes were co‐transfected with oe‐CYR61 and si‐NF‐κB p65, which displayed that the enhancement of NF‐κB p65 phosphorylation in hepatocytes by oe‐CYR61 was diminished by co‐transfection with si‐NF‐κB p65, whereas the NF‐κB p65 expression was not affected by CYR61 (*P* < 0.05) (Figure 5C). As expected, ALT and AST levels (Figure 5D), as well as contents of TNF‐α and IL‐1β (Figure 5E) in the H/R‐exposed hepatocytes, were markedly increased by the overexpression of CYR61. However, the elevated levels of these indicators were diminished by co‐transfection with si‐NF‐κB p65 in the H/R‐exposed hepatocytes (*P* < 0.05). The results of CCK‐8 (Figure 5F) and flow cytometry (Figure 5G) revealed that overexpression of CYR61 remarkably decreased viability and enhanced apoptosis of H/R‐exposed hepatocytes. By contrast, si‐NF‐κB p65 reversed the effect of oe‐CYR61 on cell viability and apoptosis (*P* < 0.05). These findings confirmed that CYR61 aggravated the H/R‐induced hepatocyte injury *via* activation of the NF‐κB signalling pathway.

### 3.6 Inhibition of NF‐κB p65 alleviates hepatocyte injury triggered by H/R through repressing TGFβ1

**FIGURE 6:**
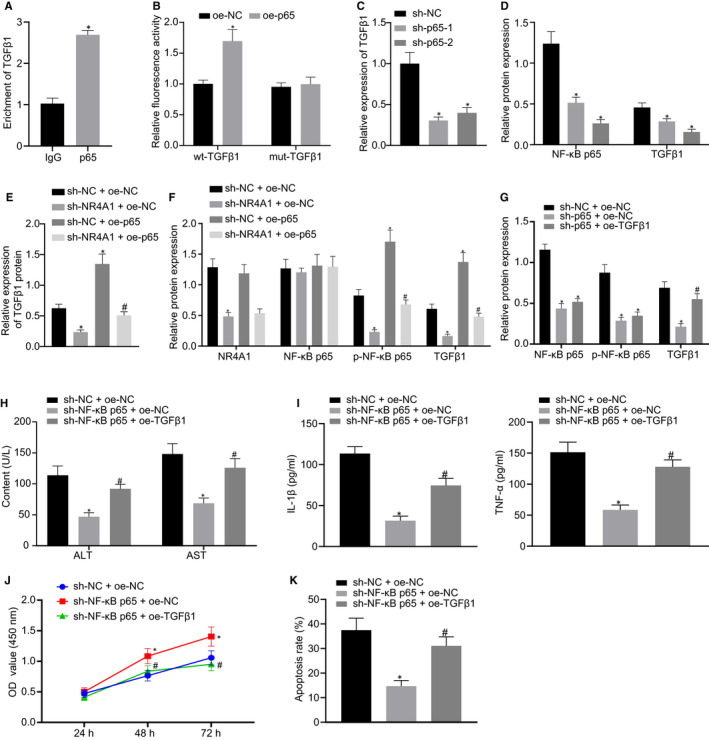
NF‐κB p65 silencing attenuates H/R‐induced hepatocyte injury through inhibiting TGFβ1. A, The binding of p65 to the TGFβ1 promoter validated through ChIP assay. ^*^ *P* < 0.05 *vs*. IgG. B, The relationship between p65 and TGFβ1 identified using dual‐luciferase reporter gene assay. ^*^ *P* < 0.05 *vs*. oe‐NC‐transfected cells. C, TGFβ1 expression in the H/R‐treated hepatocytes determined by RT‐qPCR upon NF‐κB p65 silencing. ^*^ *P* < 0.05 *vs*. si‐NC‐transfected cells. D, Protein expression of NF‐κB p65 and TGFβ1 in the H/R‐treated hepatocytes measured by Western blot analysis upon NF‐κB p65 silencing. ^*^ *P* < 0.05 *vs*. si‐NC‐transfected cells. E, The protein expression of TGFβ1 in the H/R‐treated hepatocytes measured by Western blot analysis in response to NR4A1 silencing and/or CYR61 overexpression. ^*^ *P* < 0.05 *vs*. cells co‐transfected with si‐NC and oe‐NC. ^#^ *P* < 0.05 *vs*. si‐NR4A1 and oe‐NC co‐transfected cells. F, The protein expression of NR4A1, NF‐κB p65 and TGFβ1 in the H/R‐treated hepatocytes measured by Western blot analysis in response to NR4A1 silencing and/or NF‐κB p65 overexpression. ^*^ *P* < 0.05 *vs*. cells co‐transfected with si‐NC and oe‐NC. ^#^ *P* < 0.05 *vs*. cells co‐transfected with si‐NR4A1 and oe‐NC. G, The NF‐κB p65 and TGFβ1 protein expression in the H/R‐treated hepatocytes measured using Western blot analysis. H, ALT and AST levels in the H/R‐treated hepatocytes in response to NF‐κB p65 silencing and/or TGFβ1 overexpression. I, The levels of TNF‐α and IL‐1β in the H/R‐treated hepatocytes detected by ELISA. J, Viability of the H/R‐treated hepatocytes evaluated using CCK‐8 after NF‐κB p65 silencing and/or TGFβ1 overexpression. K, Apoptosis of the H/R‐treated hepatocytes assessed by flow cytometry after NF‐κB p65 silencing and/or TGFβ1 overexpression. In panels G‐K, ^*^ *P* < 0.05 *vs*. cells co‐transfected with si‐NC and oe‐NC. ^#^ *P* < 0.05 *vs*. cells co‐transfected with si‐p65 and oe‐NC. All data were expressed as mean ± standard deviation. An unpaired t test was performed for comparisons of unpaired data between the two groups. One‐way ANOVA was conducted for comparison among multiple groups, followed by Tukey's post hoc test. Data of different groups at different time points were compared by repeated measures ANOVA, followed by Bonferroni's post hoc test. The cell experiment was repeated three times

The p65 binding site sequence on the TGFβ1 promoter was CTTCCTGGGTG through ChIPBase database (http://rna.sysu.edu.cn/chipbase/index.php). Moreover, ChIP assay was carried out to validate the binding of p65 to TGFβ1 promoter in H/R‐treated hepatocytes, whose results demonstrated that compared with the IgG group, the TGFβ1 enrichment in the p65 group was increased (*P* < 0.05) (Figure 6A). Besides, we performed a dual‐luciferase reporter assay in HEK‐293T cells to verify the binding relationship between the p65 and TGFβ1 and results of which revealed that co‐transfection of oe‐p65 resulted in a significant increase in luciferase activity of wt‐TGFβ1 (*P* < 0.05), but no difference was observed in the luciferase activity of mut‐TGFβ1 (*P* > 0.05) (Figure 6B). Intriguingly, our data from RT‐qPCR indicated that TGFβ1 mRNA expression was significantly reduced in the H/R‐treated hepatocytes after silencing of NF‐κB p65 with si‐p65‐1 and si‐p65‐2 (*P* < 0.05) (Figure 6C). The results of Western blot analysis also verified that the protein expression of NF‐κB p65 and TGFβ1 in the H/R‐treated hepatocytes was markedly down‐regulated by transfection with si‐p65‐1 and si‐p65‐2, among which si‐p65‐2 exhibited a better silencing efficacy (*P* < 0.05) (Figure 6D). Additionally, the TGFβ1 protein expression in the H/R‐treated hepatocytes was markedly decreased by silencing of NR4A1. However, the TGFβ1 protein expression was restored by the overexpression of NF‐κB p65 (*P* < 0.05) (Figure 6E). Notably, we also found that the NF‐κB p65 phosphorylation level and TGFβ1 protein expression in the H/R‐treated hepatocytes were strikingly suppressed by silencing of NR4A1, whereas NF‐κB p65 expression remained unchanged while overexpression of NF‐κB p65 resulted in increased expression of TGFβ1. Importantly, co‐transfection with oe‐p65 markedly increased the si‐NR4A1‐inhibited protein expression of TGFβ1 (*P* < 0.05) (Figure 6F). Thus, the H/R‐treated hepatocytes were co‐transfected with si‐p65 and oe‐TGFβ1 and we found that TGFβ1 protein expression was markedly decreased in the H/R‐treated hepatocytes upon NF‐κB p65 silencing. TGFβ1 expression inhibited by si‐p65 was restored by co‐transfection with oe‐TGFβ1 (*P* < 0.05) (Figure 6G). Biochemical analysis revealed that ALT and AST levels (Figure 6H) and contents of TNF‐α and IL‐1β (Figure 6I) in the H/R‐treated hepatocytes were remarkably reduced by the silencing of NF‐κB p65. However, restoration of TGFβ1 reversed the suppressive effects of si‐p65 on ALT and AST levels and contents of TNF‐α and IL‐1β (*P* < 0.05). CCK‐8 and flow cytometric analyses showed that NF‐κB p65 silencing contributed to enhanced viability and restrained the apoptosis of H/R‐treated hepatocytes. However, the effects of si‐p65 on the viability and the apoptotic rate were partially neutralized by oe‐TGFβ1 (*P* < 0.05) (Figure 6J, 6K). Taken together, the suppression of NF‐κB p65 attenuated the H/R‐induced hepatocyte injury through the down‐regulation of TGFβ1.

### 3.7 Silencing of NR4A1 relieves hepatic I/R injury in mice

**FIGURE 7:**
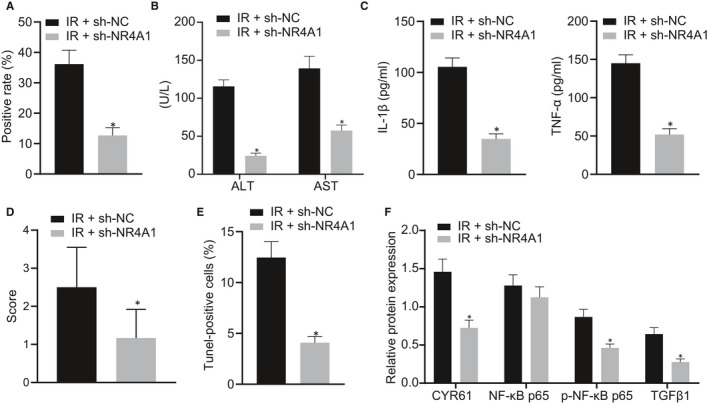
Down‐regulation of NR4A1 inhibits hepatic I/R injury in mice. A, The positive expression of NR4A1 in the hepatic tissues detected by IHC staining. B, ALT and AST levels in the serum of mice detected using biochemical analysis. C, Serum TNF‐α and IL‐1β levels detected by ELISA. D, Hepatic injury observed by HE staining. E, Apoptosis of hepatocytes was detected by TUNEL staining. F, The expression of CYR61, p‐NF‐κB p65, NF‐κB p65 and TGFβ1 in the hepatic tissues measured by Western blot analysis. ^*^ *P* < 0.05 *vs*. exposed I/R infected with lentivirus expressing sh‐NC. Data were expressed as mean ± standard deviation. An unpaired t test was performed for comparisons of unpaired data between the two groups. n = 6

To define the effect of NR4A1 on hepatic I/R injury in vivo, mice exposed to hepatic I/R were injected with lentivirus expressing sh‐NR4A1, and the positive expression of NR4A1 in the hepatic tissues was detected by IHC staining. Our data demonstrated the significantly reduced expression of NR4A1 in the I/R‐operated mice injected with sh‐NR4A1 (*P* < 0.05) (Figure 7A). As expected, ALT and AST levels (Figure 7B), as well as TNF‐α and IL‐1β levels (Figure 7C) in the serum of I/R‐operated mice were down‐regulated by the silencing of NR4A1 (*P* < 0.05). Afterwards, the hepatic tissue samples were observed by HE staining, which exhibited hepatocyte oedema, patchy necrosis, massive infiltration of neutrophils and disappearance of hepatic sinus structures in some areas of the hepatic tissues in the mice exposed to I/R. However, the above‐mentioned damage was significantly improved after the silencing of NR4A1 (Figure 7D). Further investigation of hepatocytes apoptosis by the TUNEL assay demonstrated that the silencing of NR4A1 in the I/R‐operated mice resulted in fewer TUNEL‐labelled apoptotic cells (Figure 7E). Additionally, our results from Western blot analysis exhibited that CYR61 and TGFβ1 protein expression, as well as phosphorylation of NF‐κB p65, was markedly reduced by silencing of NR4A1 (*P* < 0.05) in the I/R‐operated mice (Figure 7F). Collectively, our data demonstrated that the silencing of NR4A1 blocked the CYR61/NF‐κB/TGFβ1 axis, thereby relieving the hepatic I/R injury in mice.

## 4 DISCUSSION

I/R injury is attributed to initial hypoxia and subsequent blood flow which eventually results in organ injury.^28^ Currently, I/R injury remains a significant contributing factor that impacts morbidity and mortality following liver transplantation.^29^ In most cases, hepatic I/R injury would be generated in the course of operation on liver, and the occurrence of hepatic I/R injury is likely to cause hepatic failure.^30^ Nevertheless recently reported study has indicted initial hypoxia and subsequent blood flow as the prominent cause of I/R injury, which eventually results in organ injury.^28^ Hepatic ischaemic injury is attributed to a complex network of interactions between the diverse cellular and humoral contributors to the inflammatory response.^31^ This study mainly investigated the regulatory role of transcription factor NR4A1 in the pathophysiology of hepatic I/R injury through meditating CYR61/NF‐κB/TGFβ1 axis and provided evidence implicating that NR4A1elevates CYR61 and TGFβ1 expression to activate the NF‐κB signalling pathway, which eventually exacerbates hepatic I/R injury.

Our study revealed a significant increase of NR4A1 expression in hepatic tissues of mice with hepatic I/R injury and H/R‐exposed hepatocytes and inhibition of NR4A1 alleviates H/R‐induced hepatocyte injury by enhancing viability and reducing apoptosis of hepatocytes. Of note, NR4A1 has documented being dysregulated under hypoxia condition,^32^ which further aid in neuronal protection against oxygen and glucose deprivation‐induced damage.^33^ Moreover, the genetic ablation of NR4A1 contributes to protection against cardiac microvascular I/R injury through inhibition of FUNDC1‐mediated mitophagy.^11^ A recent study has reported that the silencing of NR4A1 elevates Mfn2 *via* the MAPK‐ERK‐CREB signalling pathway which ultimately reverses the cerebral I/R injury.^12^ Another study has demonstrated that NR4A1 knockdown ameliorates renal I/R damage *via* activating β‐catenin signalling pathway.^34^ Moreover, our study suggested that inhibition of NR4A1 protected against I/R damage in mice, supported by reductions in ALT and AST levels and down‐regulation of TNF‐α and IL‐1β. Ischaemic injury has indicated to be associated with systemic inflammation because of cytokine production and increased expression of adhesion molecules by hypoxic parenchymal and endothelial cells.^35^ Specifically, the serum levels of ALT and AST along with the expression of TNF‐α and IL‐1β are strikingly boosted in mice with hepatic I/R.^36^, ^37^ Distinguished from the studies in the past, this study further suggested a regulatory axis underlying the contributory role of NR4A1 in hepatic I/R injury.

In the subsequent study, depletion of NR4A1 relieved the hepatic injury following I/R by suppressing the expression of CYR61. Consistently, a previous study has demonstrated the co‐expression of NR4A1 and CYR61 in I/R injury.^9^ Furthermore, a markedly enhanced expression of CYR61 in the local inflammation and wound repair areas has also been reported study has demonstrated in the swine model following intestinal I/R injury.^14^ However, suppression of CYR61 suppresses inflammation and fibrosis following ischaemic kidney injury.^38^ Moreover, ectopic expression of CYR61 induced hepatic stellate cell apoptosis.^39^ Intriguingly, our study illustrated that suppression of CYR61 mitigated I/R‐induced hepatic damage and H/R‐triggered hepatocellular injury through inhibiting apoptosis of hepatocytes.

Moreover, NF‐κB expression has illustrated being highly elevated in liver injury triggered by I/R,^16^ whereas suppression of NF‐κB relieves hepatic I/R injury and decreases apoptosis of hepatocytes.^40^ Moreover, overexpression of CYR61 has been shown to contribute to the enhancement of NF‐κB p65.^26^ Consistently, we found that CYR61 could activate NF‐κB signalling pathway, hence promoting the apoptosis of hepatocytes. In the light of a prior report, overexpression of CYR61 contributes to enhancement of NF‐κB p65.^26^ NF‐κB expression is highly elevated in liver injury triggered by I/R,^16^ whereas repression of NF‐κB relieves hepatic I/R injury and decreases apoptosis of hepatocytes.^40^ It has consistently been reported that activation of transcription factor NF‐κB in Kupffer cells aggravates inflammatory response in mice with hepatic I/R injury.^41^ Similarly, the present study demonstrated that the silencing of NF‐κB p65 inhibited hepatocellular injury *via* suppressing the expression of TGFβ1. It has been further supported by a previous finding that induction of TGFβ1 mainly depends on the activation of NF‐κB, whereas TGFβ1 contributes to the degradation of the NF‐κB inhibitor IκBα and promotes the nuclear translocation of the NF‐κB p65 subunit.^19^ Moreover, inhibition of TGFβ1 has also been reported to suppress the myocardial cell apoptosis following I/R.^21^ NR4A1 knockdown could restrain cerebral ischaemia‐induced neuroinflammation and alleviate cerebral damage *via* NF‐κB p65.^42^ Our in vitro and in vivo experiments also suggested that NR4A1 knockdown led to inhibition of CYR61, NF‐κB and TGFβ1, hence diminishing hepatic I/R injury.

**FIGURE 8:**
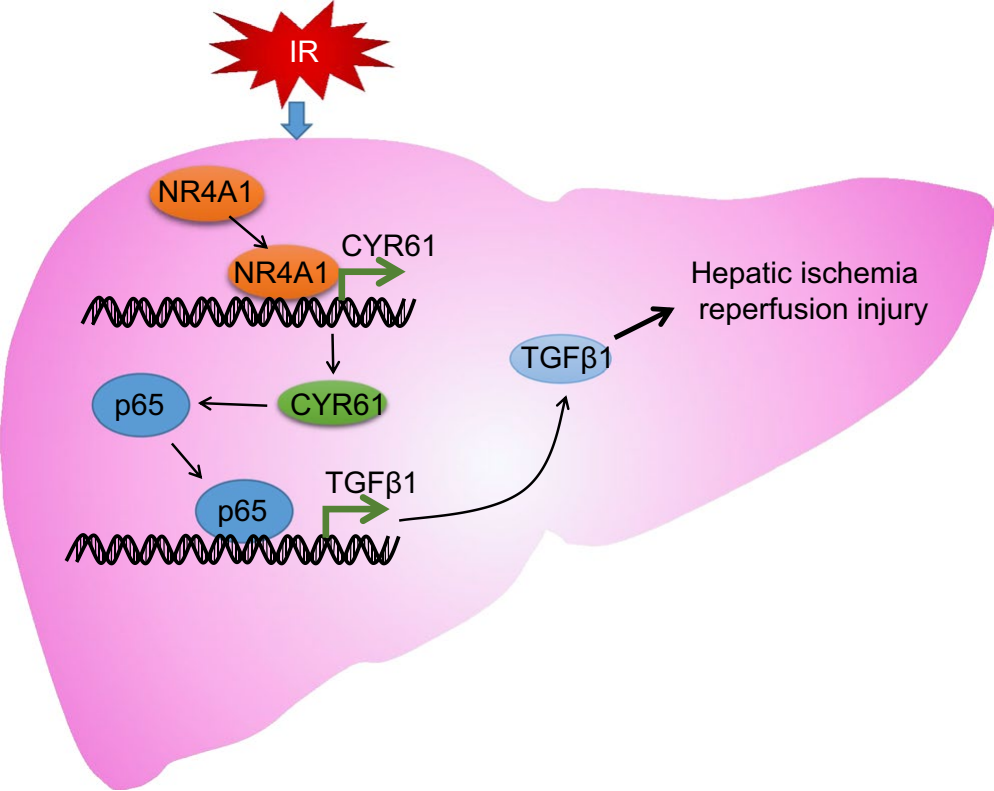
Molecular mechanism of NR4A1 implicated in the hepatic I/R injury. NR4A1, highly expressed in hepatic I/R injury, elevates CYR61 expression and activates the NF‐κB signalling pathway. Afterwards, NF‐κB p65 binds to the TGFβ1 promoter to activate TGFβ1 transcription, thus eventually exacerbating hepatic I/R injury

Conclusively, the above‐described findings supported our hypothesis that silencing of NR4A1 inhibits CYR61, NF‐κB and TGFβ1 expression, thereby attenuating hepatic I/R injury (Figure 8). Hence, the present study provides a novel concept for the mechanisms involved in the pathogenesis of hepatic I/R injury and highlights promising therapeutic targets for hepatic I/R injury. In the near future, these laboratory findings are expected to be translated from bench to bedside.

## CONFLICTS OF INTERESTS

All authors declare that they have no conflicts of interests.

## AUTHOR CONTRIBUTION

**Jun Cao:** Conceptualization (equal); Formal analysis (equal). **Ting Xu:** Formal analysis (equal); Investigation (equal). **Chengming Zhou:** Supervision (equal); Writing‐original draft (equal). **Shaochuang Wang:** Project administration (equal); Writing‐review & editing (equal). **Baofei Jiang:** Data curation (equal); Resources (equal). **Kun Wu:** Validation (equal); Writing‐original draft (equal). **Long Ma:** Funding acquisition (equal); Methodology (equal).

## Data Availability

Research data are not shared.

## References

[1] Eltzschig HK , Eckle T . Ischemia and reperfusion–from mechanism to translation. Nat Med. 2011;17:1391‐1401. 22064429 10.1038/nm.2507 PMC3886192

[2] Peralta C , Jimenez‐Castro MB , Gracia‐Sancho J . Hepatic ischemia and reperfusion injury: effects on the liver sinusoidal milieu. J Hepatol. 2013;59:1094‐1106. 23811302 10.1016/j.jhep.2013.06.017

[3] Jimenez‐Castro MB , Cornide‐Petronio ME , Gracia‐Sancho J , Peralta C . Inflammasome‐Mediated Inflammation in Liver Ischemia‐Reperfusion Injury. Cells. 2019;8(10):1131. 10.3390/cells8101131 PMC6829519 31547621

[4] Yang M , Antoine DJ , Weemhoff JL , et al. Biomarkers distinguish apoptotic and necrotic cell death during hepatic ischemia/reperfusion injury in mice. Liver Transpl. 2014;20:1372‐1382. 25046819 10.1002/lt.23958 PMC4213307

[5] Yang W , Chen J , Meng Y , Chen Z , Yang J . Novel targets for treating ischemia‐reperfusion injury in the liver. Int J Mol Sci. 2018;19(5):1302. 10.3390/ijms19051302 PMC5983804 29701719

[6] Wang X , Mao W , Fang C , et al. Dusp14 protects against hepatic ischaemia‐reperfusion injury via Tak1 suppression. J Hepatol. 2018;68(1):118–129. 10.1016/j.jhep.2017.08.032 28887166

[7] Weigand K , Brost S , Steinebrunner N , et al. Ischemia/Reperfusion injury in liver surgery and transplantation: pathophysiology. HPB Surg. 2012;2012:176723. 22693364 10.1155/2012/176723 PMC3369424

[8] Lambert SA , Jolma A , Campitelli LF , et al. The Human Transcription Factors. Cell. 2018;172:650‐665. 29425488 10.1016/j.cell.2018.01.029 PMC12908702

[9] Zhang P , Ming Y , Cheng K , Niu Y , Ye Q . Gene expression profiling in ischemic postconditioning to alleviate mouse liver ischemia/reperfusion injury. Int J Med Sci. 2019;16:343‐354. 30745817 10.7150/ijms.29393 PMC6367534

[10] Ohkubo T , Sugawara Y , Sasaki K , et al. Early induction of nerve growth factor‐induced genes after liver resection‐reperfusion injury. J Hepatol. 2002;36:210‐217. 11830332 10.1016/s0168-8278(01)00258-6

[11] Zhou H , Wang J , Zhu P , et al. NR4A1 aggravates the cardiac microvascular ischemia reperfusion injury through suppressing FUNDC1‐mediated mitophagy and promoting Mff‐required mitochondrial fission by CK2alpha. Basic Res Cardiol. 2018;113:23. 29744594 10.1007/s00395-018-0682-1

[12] Zhang Z , Yu J . NR4A1 promotes cerebral ischemia reperfusion injury by repressing Mfn2‐mediated mitophagy and inactivating the MAPK‐ERK‐CREB signaling pathway. Neurochem Res. 2018;43:1963‐1977. 30136162 10.1007/s11064-018-2618-4

[13] Klingenberg R , Aghlmandi S , Liebetrau C , et al. Cysteine‐rich angiogenic inducer 61 (Cyr61): a novel soluble biomarker of acute myocardial injury improves risk stratification after acute coronary syndromes. Eur Heart J. 2017;38:3493‐3502. 29155984 10.1093/eurheartj/ehx640

[14] Shegarfi H , Krohn CD , Gundersen Y , et al. Regulation of CCN1 (Cyr61) in a porcine model of intestinal ischemia/reperfusion. Innate Immun. 2015;21:453‐462. 25783840 10.1177/1753425915569089

[15] Zhu X , Song Y , Wu C , et al. Cyr61 participates in the pathogenesis of acute lymphoblastic leukemia by enhancing cellular survival via the AKT/NF‐kappaB signaling pathway. Sci Rep. 2016;6:34018. 27725691 10.1038/srep34018 PMC5057070

[16] Zhu J , Zhu F , Song W , et al. Altered miR‐370 expression in hepatic ischemia‐reperfusion injury correlates with the level of nuclear kappa B (NF‐kappaB) related factors. Gene. 2017;607:23‐30. 28043920 10.1016/j.gene.2016.12.026

[17] Ramachandran S , Liaw JM , Jia J , et al. Ischemia‐reperfusion injury in rat steatotic liver is dependent on NFkappaB P65 activation. Transpl Immunol. 2012;26:201‐206. 22286145 10.1016/j.trim.2012.01.001 PMC3675789

[18] Huang Z , Zheng D , Pu J , et al. MicroRNA‐125b protects liver from ischemia/reperfusion injury via inhibiting TRAF6 and NF‐kappaB pathway. Biosci Biotechnol Biochem. 2019;83:829‐835. 30686117 10.1080/09168451.2019.1569495

[19] Madhyastha R , Madhyastha H , Pengjam Y , et al. NFkappaB activation is essential for miR‐21 induction by TGFbeta1 in high glucose conditions. Biochem Biophys Res Commun. 2014;451:615‐621. 25130469 10.1016/j.bbrc.2014.08.035

[20] Nepon‐Sixt BS , Alexandrow MG . TGFbeta1 cell cycle arrest is mediated by inhibition of MCM assembly in Rb‐deficient conditions. Mol Cancer Res. 2019;17:277‐288. 30257992 10.1158/1541-7786.MCR-18-0558 PMC6318023

[21] Liu YF , Chu YY , Zhang XZ , et al. TGFbeta1 protects myocardium from apoptosis and oxidative damage after ischemia reperfusion. Eur Rev Med Pharmacol Sci. 2017;21:1551‐1558. 28429351

[22] Fan XD , Zheng HB , Fan XS , Lu S . Increase of SOX9 promotes hepatic ischemia/reperfusion (IR) injury by activating TGF‐beta1. Biochem Biophys Res Commun. 2018;503:215‐221. 29879429 10.1016/j.bbrc.2018.06.005

[23] Gu N , Ge K , Hao C , et al. Neuregulin1beta effects on brain tissue via ERK5‐dependent MAPK pathway in a rat model of cerebral ischemia‐reperfusion injury. J Mol Neurosci. 2017;61:607‐616. 28265860 10.1007/s12031-017-0902-4

[24] Coulouarn C , Factor VM , Thorgeirsson SS . Transforming growth factor‐beta gene expression signature in mouse hepatocytes predicts clinical outcome in human cancer. Hepatology. 2008;47:2059‐2067. 18506891 10.1002/hep.22283 PMC2762280

[25] Suzuki S , Toledo‐Pereyra LH , Rodriguez FJ , Cejalvo D . Neutrophil infiltration as an important factor in liver ischemia and reperfusion injury. Modulating effects of FK506 and cyclosporine. Transplantation. 1993;55:1265‐1272. 7685932 10.1097/00007890-199306000-00011

[26] Cao Y , Wu C , Song Y , et al. Cyr61 decreases Cytarabine chemosensitivity in acute lymphoblastic leukemia cells via NF‐kappaB pathway activation. Int J Mol Med. 2019;43:1011‐1020. 30535449 10.3892/ijmm.2018.4018

[27] Bian L , Zhi X , Ma L , et al. Hsa_circRNA_103809 regulated the cell proliferation and migration in colorectal cancer via miR‐532‐3p / FOXO4 axis. Biochem Biophys Res Commun. 2018;505:346‐352. 30249393 10.1016/j.bbrc.2018.09.073

[28] Nace GW , Huang H , Klune JR , et al. Cellular‐specific role of toll‐like receptor 4 in hepatic ischemia‐reperfusion injury in mice. Hepatology. 2013;58:374‐387. 23460269 10.1002/hep.26346 PMC3688695

[29] Ueki S , Castellaneta A , Yoshida O , et al. Hepatic B7 homolog 1 expression is essential for controlling cold ischemia/reperfusion injury after mouse liver transplantation. Hepatology. 2011;54:216‐228. 21503939 10.1002/hep.24360 PMC3125416

[30] Sun P , Zhang P , Wang PX , et al. Mindin deficiency protects the liver against ischemia/reperfusion injury. J Hepatol. 2015;63:1198‐1211. 26165142 10.1016/j.jhep.2015.06.033

[31] Abu‐Amara M , Yang SY , Tapuria N , et al. Liver ischemia/reperfusion injury: processes in inflammatory networks–a review. Liver Transpl. 2010;16:1016‐1032. 20818739 10.1002/lt.22117

[32] Huang HM , Yu JY , Ou HC , Jeng KC . Effect of naloxone on the induction of immediately early genes following oxygen‐ and glucose‐deprivation in PC12 cells. Neurosci Lett. 2008;438:252‐256. 18457920 10.1016/j.neulet.2008.04.036

[33] Xiao G , Sun T , Songming C , Cao Y . NR4A1 enhances neural survival following oxygen and glucose deprivation: an in vitro study. J Neurol Sci. 2013;330:78‐84. 23663895 10.1016/j.jns.2013.04.010

[34] Shi W , Dong J , Liang Y , Liu K , Peng Y . NR4A1 silencing protects against renal ischemia‐reperfusion injury through activation of the beta‐catenin signaling pathway in old mice. Exp Mol Pathol. 2019;111:104303. 31465766 10.1016/j.yexmp.2019.104303

[35] Soares ROS , Losada DM , Jordani MC , Evora P , Castro‐e‐Silva O . Ischemia/Reperfusion injury revisited: an overview of the latest pharmacological strategies. Int J Mol Sci. 2019;20(20):5034. 10.3390/ijms20205034 PMC6834141 31614478

[36] Perry BC , Soltys D , Toledo AH , Toledo‐Pereyra LH . Tumor necrosis factor‐alpha in liver ischemia/reperfusion injury. J Invest Surg. 2011;24:178‐188. 21675854 10.3109/08941939.2011.568594

[37] Liu P , Xu B , Spokas E , Lai PS , Wong PY . Role of endogenous nitric oxide in TNF‐alpha and IL‐1beta generation in hepatic ischemia‐repefusion. Shock. 2000;13:217‐223. 10718379 10.1097/00024382-200003000-00008

[38] Lai CF , Lin SL , Chiang WC , et al. Blockade of cysteine‐rich protein 61 attenuates renal inflammation and fibrosis after ischemic kidney injury. Am J Physiol Renal Physiol. 2014;307:F581‐F592. 24920753 10.1152/ajprenal.00670.2013

[39] Borkham‐Kamphorst E , Steffen BT , Van de Leur E , et al. CCN1/CYR61 overexpression in hepatic stellate cells induces ER stress‐related apoptosis. Cell Signal. 2016;28:34‐42. 26515130 10.1016/j.cellsig.2015.10.013

[40] Liu A , Huang L , Fan H , et al. Baicalein pretreatment protects against liver ischemia/reperfusion injury via inhibition of NF‐kappaB pathway in mice. Int Immunopharmacol. 2015;24:72‐79. 25479717 10.1016/j.intimp.2014.11.014

[41] Sakai N , Van Sweringen HL , Schuster R , et al. Receptor activator of nuclear factor‐kappaB ligand (RANKL) protects against hepatic ischemia/reperfusion injury in mice. Hepatology. 2012;55:888‐897. 22031462 10.1002/hep.24756 PMC3276725

[42] Zhang YJ , Song JR , Zhao MJ . NR4A1 regulates cerebral ischemia‐induced brain injury by regulating neuroinflammation through interaction with NF‐kappaB/p65. Biochem Biophys Res Commun. 2019;518:59‐65. 31445702 10.1016/j.bbrc.2019.08.008

